# Impact of Common and Rare *APOE* Missense Variants on Alzheimer's Disease and Related Traits in Asian Populations

**DOI:** 10.1002/alz70855_104939

**Published:** 2025-12-24

**Authors:** Donghe Li, Sarang Kang, Kun Ho Lee, Gyungah R Jun

**Affiliations:** ^1^ Boston University Chobanian & Avedisian School of Medicine, Boston, MA, USA; ^2^ Gwangju Alzheimer's and Related Dementia (GARD) Cohort Research Center, Chosun University, Gwangju, Korea, Republic of (South); ^3^ Gwangju Alzheimer's Disease and Related Dementias (GARD) Cohort Research Center, Gwangju, Korea, Republic of (South); ^4^ Korea Brain Research Institute, Daegu, Korea, Republic of (South); ^5^ Biomedical Science, Chosun University, Gwangju, Gwangju, Korea, Republic of (South); ^6^ Biomedical Genetics Section, Department of Medicine, Boston University Chobanian & Avedisian School of Medicine, Boston, MA, USA; ^7^ Alzheimer's Disease Research Center, Boston University Chobanian & Avedisian School of Medicine, Boston, MA, USA

## Abstract

**Background:**

APOE is a well‐known susceptibility gene for Alzheimer's disease (AD), with common alleles (APOE2, 3, and 4) extensively studied across ancestries. Recent studies identified significant associations between rare missense variants (RMVs) and AD in Caucasian populations, with unique RMVs reported in Japanese cohorts. Investigating the roles of common and rare APOE variants in AD and related traits in East Asians is important.

**Method:**

We analyzed data from the Gwangju Alzheimer's and Related Dementias (GARD) cohort, consisting of over 16,000 participants with up to 10 years of follow‐up in South Korea. APOE common genotypes (E3/3 as reference) and allelic doses (E2 and E4) were evaluated for associations with AD, cognitive traits, and brain imaging (MRI) measures, adjusting for age and sex. We will investigate the impact of APOE RMVs, including previously reported and Asian‐specific variants, on AD and related traits. Meta‐analyses will include Asian participants from large cohorts such as Asian Cohort for Alzheimer's Disease (ACAD), Alzheimer's Disease Genetics Consortium (ADGC), and UK Biobank (UKBB).

**Result:**

After excluding MCI cases, 7,389 participants (1,949 AD cases, 5,440 controls) were analyzed. Odds ratios (ORs) increased with fewer E2 alleles and more E4 alleles, ranging from 0.65 to 9.15 compared to E3/3 genotypes. Allelic dose analysis showed a stronger effect for E4 (OR: 2.36) than E2 (OR: 0.83). Increased E4 alleles were associated with reduced hippocampal volume and entorhinal cortex thickness, while E2 showed no significant effects. Cognitive domains, including attention, visuospatial, memory, and frontal functions, declined with increasing E4 alleles but showed no significant associations with E2.

**Conclusion:**

APOE genotypes and allelic doses significantly influence AD and related traits. Comprehensive analysis of common and rare APOE variants, particularly in Asian populations with distinct risk profiles, is critical for understanding, treating, and preventing AD.

